# Hyaluronic Acid/Collagen Nanofiber Tubular Scaffolds Support Endothelial Cell Proliferation, Phenotypic Shape and Endothelialization

**DOI:** 10.3390/nano11092334

**Published:** 2021-09-08

**Authors:** Yuqing Niu, Massimiliano Galluzzi

**Affiliations:** 1 Department of Pediatric Surgery, Guangdong Provincial Key Laboratory of Research in Structural Birth Defect Disease, Guangzhou Women and Children’s Medical Center, Guangzhou Medical University, Guangzhou 510623, China; 2 Materials Interfaces Center, Shenzhen Institutes of Advanced Technology, Chinese Academy of Sciences, Shenzhen 518055, China; galluzzi@siat.ac.cn

**Keywords:** collagen, hyaluronic acid, vascular endothelial cells, nanofibers, endothelization

## Abstract

In this study, we designed and synthetized artificial vascular scaffolds based on nanofibers of collagen functionalized with hyaluronic acid (HA) in order to direct the phenotypic shape, proliferation, and complete endothelization of mouse primary aortic endothelial cells (PAECs). Layered tubular HA/collagen nanofibers were prepared using electrospinning and crosslinking process. The obtained scaffold is composed of a thin inner layer and a thick outer layer that structurally mimic the layer the intima and media layers of the native blood vessels, respectively. Compared with the pure tubular collagen nanofibers, the surface of HA functionalized collagen nanofibers has higher anisotropic wettability and mechanical flexibility. HA/collagen nanofibers can significantly promote the elongation, proliferation and phenotypic shape expression of PAECs. In vitro co-culture of mouse PAECs and their corresponding smooth muscle cells (SMCs) showed that the luminal endothelialization governs the biophysical integrity of the newly formed extracellular matrix (e.g., collagen and elastin fibers) and structural remodeling of SMCs. Furthermore, in vitro hemocompatibility assays indicated that HA/collagen nanofibers have no detectable degree of hemolysis and coagulation, suggesting their promise as engineered vascular implants.

## 1. Introduction

Cardiovascular diseases are considered as the major cause of death worldwide [1]. Artery bypass grafting or replacement is an effective method for the treatment of degenerative arterial diseases, such as massive vascular occlusions caused by atherosclerosis. In recent years, various materials, such as polytetrafluoroethylene, polycaprolactone and decellularized extracellular matrix (ECM), have been used to prepare vascular grafts [2,3]. However, due to the incomplete coverage of endothelial cells (ECs) on the surface of vascular substitutes (causing intimal hyperplasia), tissue-engineered grafts for small vessels often cause occlusions (thrombosis) [4]. Complete endothelialization along the inner wall of a scaffold holds promises in solving this issue [5,6]. Using such a method, the in vitro expanded patient-derived ECs are firstly seeded on the luminal surface of the scaffold, and allowed to form a monolayer of evenly distributed ECs prior to transplantation.

The inner blood contact layer of native blood vessels is lined with a monolayer of ECs, which adhere to the basement membrane bed [7]. The ECM of the basement membrane consists mainly of elastin, collagen, laminin and heparan sulfate proteoglycan [8]. ECs and the correspondent ECM of basement membrane form the vascular endothelium, which can maintain antithrombotic properties and inhibit intimal hyperplasia.

From the perspective of vascular tissue engineering, biomaterial scaffolds have been regarded just as a structural framework, but now they are considered as important regulators of cell phenotype and function [9,10]. As the main component of natural ECM, collagen has been widely used as a scaffold material for tubular or hollow organs due to its wide availability, good biocompatibility and easy processability [11,12,13]. Collagen-based scaffolds fabricated by electrospinning technology can create a hierarchical nano-topography and microstructure similar to native ECM [14,15,16]. The greatest advantage of collagen as a scaffold material is to guide tissue regeneration by its temporary ECM function [17,18]. However, collagen scaffolds usually have limited anticoagulant activity and poor biological activity to regulate EC response [19,20]. Therefore, the endothelialization process in collagen-based scaffold grafts is relatively slow. Studies have shown that hyaluronic acid (HA) has double functionality as anticoagulant and promoter of endothelialization [21,22,23]. HA, as a unique glycosaminoglycan, widely exists in the ECM of various tissues [10,24,25]. It has been recognized that HA plays a key role in adjusting biological functions by binding to specific protein receptors (such as CD44) on different cell surfaces [26,27]. Therefore, it is a feasible strategy to integrate HA onto the surface of collagen scaffolds, regulating cell behavior and promoting blood compatibility and endothelialization of vascular transplantation.

Herein, tubular HA/collagen nanofibrous scaffolds were obtained by sequential electrospinning and crosslinking methods. The resultant scaffolds consisted of a thin inner layer and a thick outer layer, which, respectively, simulate the intima and middle layer of natural blood vessels. HA-functionalized collagen nanofiber on the inner wall of the tube was helpful to optimize the surface biochemistry, while the layered tubular bulk architecture provided mechanical support to cells. This allowed us to hypothesize that tubular HA/collagen nanofibrous scaffolds can have a favorable effect on the phenotypic shape, spatial distribution and behavior patterns of vascular ECs.

To verify the above hypothesis, we report the preparation, physicochemical properties and blood compatibility of HA/collagen layered nanofiber tubes. On this basis, the adhesion, elongation, proliferation and phenotypic shape of mouse primary aortic endothelial cells (PAECs) on HA/collagen nanofibers were studied. Finally, PAECs and their corresponding smooth muscle cells (SMCs) were sequentially seeded into the inner and outer layers of the layered nanofiber tubes. Histochemistry and immunofluorescence were employed to evaluate the morphology, phenotype and matrix secretion of the two types of primary cells in the layered nanofiber tubes.

## 2. Materials and Methods

### 2.1. Materials and Reagents

Hyaluronic acid (Mw ≈ ~800 kDa, F1177), collagen (11179179001), plasminogen (341578), Dulbecco’s modified Eagle’s medium (DMEM, D0819), 4′6-diamidino-2′-phenylindole dihydrochloride (DAPI, 10236276001), phosphate buffered saline (PBS, pH 7.4, P4417), bovine serum albumin (BSA, A3858), paraffin (327204), and dehydrated alcohol (1012772) were obtained from Sigma-Aldrich (St. Louis, MO, USA). Fetal bovine serum (FBS, 16140071), penicillin/streptomycin (P/S, 15070063), trypsin (0.25%, 15050065), primary antibody to CD31 (37-0700), Rhodamine Phalloidin (R415) were obtained from Gibco (Grand Island, NY, USA). Anti-alpha (ɑ)-smooth muscle actin (ɑ-SMA, ab5694) and Cell Counting Kit-8 (CCK-8, ab228554) were purchased from Abcam (Cambridge, UK). The 4% paraformaldehyde (C104190), Masson’s trichrome (MTC), Verhoeff-Van Gieson (VVG, GP1035), Triton X-100 (WGT8200), hematoxylin eosin (H&E, G1004, G1002), secondary antibodies, Alexa Fluor-488, or Cy3-conjugated anti-mouse or anti-rabbit immunoglobulin-G (IgG) for fluorescence staining were obtained from Servicebio Science and Technology Co., Ltd (Wuhan, China). Hexafluoroisopropanol (HFIP, 920-66-1) was purchased from Aladdin (Shanghai, China). Mouse PAECs (SNP-M016) and SMCs (SNP-M015) were obtained from Sunncell Bioscience Inc. (Wuhan, China).

### 2.2. Preparation of HA/Collagen Nanofiber Scaffolds

Collagen (10%) and HA (2%) electrospinning solutions were prepared, respectively, by dissolving 1 g of collagen and 0.2 g of HA in 10 mL of HFIP. Firstly, 2% HA electrospinning solution was transferred into a 10 mL syringe and fixed on the precise injection pump of the electrospinning machine. The feeding rate of the solution was set at 0.36 mL/h. The voltage power supply was set at 11 kV, and the distance between the spinneret tip and the grounded drum collector was fixed at 20 cm. For comparison, collagen nanofiber films and tubular structures with similar parameters were prepared. The device collecting nanofiber in tubular structure was a stainless-steel tube with outer diameter of 3 mm, while thin films composed of nanofibers were collected on a stainless-steel rod with an outer diameter of 10 cm. Drum speed was set at 1200 rpm. The electrospinning environment was maintained at 18–25 °C and 40–45% (relative humidity). In order to obtain layered nanofiber tubes, the spinning time of electrospinning solution HA was 20 h, followed by collagen spinning solution for 40 h with the same electrospinning parameters. The spinning time of HA/collagen film was firstly set at 2 and 8 h, respectively.

### 2.3. Crosslinking of Nanofibers

Glutaraldehyde (0.1 mol/L) and hydrochloric acid (0.01 mol/L) were used to crosslink the collagen and HA/collagen nanofibers in acetone (80%) water mixture at 25 °C for 24 h. Before further use, all electrospun nanofibers were placed in a vacuum drying oven for 24 h at 25 °C to remove organic solvents.

### 2.4. Characterizations

The morphology of electrospun collagen and HA/collagen nanofibers before and after crosslinking treatment was observed by scanning electron microscopy (SEM, SU8010, Hitachi, Tokyo, Japan) with an accelerating voltage of 5 kV after sputter coating with gold [28]. Image analysis software ImageJ (http://imagej.net/citing) (accessed on 22 April 2017) was used to measure the average diameter and pore size distribution of electrospun nanofibers from SEM images. For each sample, 120 nanofibers or pores were randomly selected from 10000× magnification SEM images [29]. The fiber diameter and pore diameter were calculated by averaging 120 random nanofibers and pores, respectively.

The wettability of electrospun collagen and HA/collagen nanofibers was measured by water contact angle method (SL600, Solon Information Technology Co., Inc. (Solon, OH, USA). In short, after 2 s, 0.2 μL deionized water was dropped on the flat nanofiber membrane to take, then a static image of the water contact angle was measured perpendicularly to the nanofiber axis.

By measuring the resistance of nanofibers to plasminogen (human plasma fibrinolytic enzyme), the degradation properties of cross-linked electrospun nanofibers were measured. The initial weight (w_0_) of electrospun nanofibers before treatment with plasminogen was recorded. Then, the samples were immersed in the digestive solution containing plasminogen (25 mg/mL) (pH 7.40) and incubated at 37 °C. The plasminogen solution was refreshed every three days. Samples were removed from the digestive solution and lyophilized at each time point (day 7, 14, 21 and 28). Finally, the residual mass (w_t_) at each time point was recorded and the weight loss percentage (%) of the stent was calculated according to the following formula:

Weight loss percentage (%) = (w_0_ − w_t_/w_0_)100%

Each nanofiber film was characterized by Fourier transform infrared spectroscopy (FTIR) in the range of 500–4000 cm^−1^ and attenuated total reflection (ATR) head (ATR-FTIR, thermo Nicolet, Waltham, MA, USA) with a scanning resolution of 2 cm^−1^.

X-ray photoelectron spectroscopy (XPS) was performed using an ESCA-LAB 250Xi (Thermo Fisher, Waltham, MA, USA) to determine the surface chemistry of each nanofiber film, for which full (pass energy, 100 eV) and high-resolution spectra (pass energy, 20 eV) were recorded [30].

Wide-angle X-ray scattering (WAXS) measurements were performed using Cu k-alpha radiation (λ = 0.15418 nm) on an X-ray diffractometer (Bruker, D8 Advance, Billerica, MA, USA) equipped with an image plate detector and a graphite monochromator, and controlled by a fast/XRD diffractometer control software (RIGAKU America Corp. version 2.3.8, Tokyo, Japan). Fix the nanofiber sample vertically on the copper sample table. The diffraction data were collected for 20 min while the sample stage was rotated at a speed of 5° min^−1^.

To ensure that the electrospun collagen and HA/collagen nanofiber scaffolds can withstand pressure changes in blood vessels, their ultimate tensile strength (UTS) and linear modulus (E) values were measured by a tensile testing instrument (Sans, Shenzhen, China). Rectangular specimens (60 × 12 × 0.10–0.15 mm) were held between the instrument grids with a 50 N load cell, and we conducted a fracture test at a strain rate of 2 mm/min with an initial holding distance of 60 mm. All measurements were performed at room temperature (RT). The tensile properties were obtained from the initial linear region of the stress–strain curve of the specimen [30,31]. Five samples were tested for each type of sample.

### 2.5. In Vitro Hemocompatibility Evaluation

#### 2.5.1. Red Blood Cell (RBC) Morphology on Nanofibers

The animal-related experiment protocol was approved by the animal experimentation Ethics committee of Guangzhou Medical University. The collagen and HA/collagen nanofibers were cut into circular membranes with a diameter of 15 mm. The as-prepared nanofiber circular membranes were inserted in a 24-well plate, following addition of 70% ethanol solution to sterilize the nanofiber samples at room temperature (RT) for 2 h, and finally pre-warmed 1× PBS to rinse the sample 3 times for further use. A 4 mL blood quantity was sampled from healthy New Zealand white rabbits and trisodium citrate was employed for anticoagulation at a volume ratio of 9:1. After 1500 rpm centrifugation for 10 min at 4 °C, the supernatant was removed. The RBCs were washed with 1× PBS, and incubated with different nanofiber samples in 1× PBS for 60 min at RT. After that, the RBCs were fixed with 4% formaldehyde for 15 min, and dehydrated with ethanol gradient solutions (85%, 95% and 100%, *v*/*v*). After freeze-drying, the RBC nanofiber scaffolds were plated with gold and observed with SEM.

#### 2.5.2. RBC Lysis

The hemolysis rate was evaluated by determining the hemoglobin concentration released by diluted red blood cells (RBC) exposed to the electrospun nanofiber material. The negative control consisted of adding 50 μL of RBC suspension (16% in 1× PBS, *v*/*v*) to a centrifuge tube in 2 mL 1× PBS (as a negative control). As a positive control, 2 mL deionized water was used in place of 1× PBS in order to induce maximum red blood cell lysis (*n* = 4). After incubating for a selected period of time, the RBC suspension was centrifuged at 2000 rpm for 5 min, and the supernatant was collected. Then, a microplate reader (Multiskan MK33, Thermo electron corporation, China) was used to measure the absorbance of hemoglobin (HB) released in the supernatant (200 μL) at 540 nm. By comparing the absorbance values of the tested supernatant and the positive control (i.e., 100% hemolysis), the hemolysis rate in the presence of different nanofibers was calculated.

#### 2.5.3. Platelet Adhesion Test

Platelet-rich plasma (PRP) was obtained from the above-mentioned whole blood separated by centrifugation and used for the platelet adhesion test. A 100 microliters quantity of PRP was added to each well plate covered with nanofiber film samples, and then incubate for 2 h at 37 °C. Then, specimens were rinsed gently with pre-warmed 1× PBS twice, followed by subsequent fixation, gradient dehydration, freeze-drying, and SEM observation.

#### 2.5.4. Activated Partial Thromboplastin Time (APTT) and Prothrombin Time (PT) Evaluation

Fresh anticoagulated whole blood was centrifuged at 1500 rpm/min for 10 min, and the supernatant (platelet-deficient plasma) was collected. The platelet-poor plasma (270 μL) was added to the nanofiber-covered well plate. According to the instructions of the reagent manufacturer, after adding the corresponding reagents, the APTT and PT of the samples were measured with an automatic coagulation analyzer (H1201, Jiansu Horner Medical Instrument Co., Ltd., Changshu, China).

### 2.6. In Vitro Cell Scaffold Interactions

Mouse PAECs (passage 3) were cultured in DMEM containing 10% FBS, 1% P/S (penicillin/streptomycin) in a 37 °C, 5% CO_2_ humidified incubator. Cells were seeded on nanofibers at a concentration of 1 × 10^5^ cells/cm^2^, and the medium was changed every two days. Before cell seeding, each crosslinked nanofiber sample was sterilized with 70% ethanol for 2 h, and then washed with 1× PBS 5 times. The tissue culture plate (TCP, in vitro conventional two-dimensional cell culture mode) was set as the control group.

At 12 h seeding, the sterilized nanofiber film samples (15 mm in diameter) were added in to each pore of the TCP and cultured for 24 and 48 h followed by staining using a live/dead assay kit (L3224, Invitrogen) using previously described methods [32,33]. In short, after sucking and discarding the culture medium, we washed the cells and nanofiber film samples in each well plate well with 1× PBS, then added 1 mL staining solution containing 0.4 μL Calcein AM and 2 μL ether-homodimer-1 to each well, and incubated in the dark for 30 min. A confocal laser scanning microscope (CLSM, TCS SP5, Leica, Wetzlar, Germany) observed cell staining, with the excitation/emission filter set at 488/530 nm to observe living cells (stained green), and detected dead cells (stained red) at 530/580 nm. Five images were randomly selected from each hole containing nanofiber film samples for shooting and analysis. Wells without nanofiber film samples were set as positive controls.

At 24 and 36 h seeding, the cell growth and morphology on different substrates were studied by SEM. Briefly, the cell scaffold samples were washed twice with 1× PBS to remove non-adherent cells, and fixed with 4% paraformaldehyde at RT for 15 min. Then, samples were rinsed with 1× PBS 3 times, followed by a series of ethanol solution gradient dehydration, freeze-drying, and SEM observation.

The proliferation of mouse PAECs on each nanofiber and TCP (positive control) was analyzed using a CCK-8 kit at time points 24, 48, 72, 96 and 120 h after seeding, as described previously [34].

### 2.7. Cell Phenotype Analysis

A total of 72 h after seeding, the cells’ phenotype and morphology were studied by immunofluorescence. Briefly, the cell scaffold samples were fixed with 4% paraformaldehyde for 15 min at RT. Subsequently, cells were permeabilized with 0.1% Triton X-100 for 15 min, and blocked with 1% BSA for 30 min. The cell scaffold samples were treated with primary antibody CD31 diluted 1:150 at 4 °C overnight. Then, Alexa Fluor-488-conjugated anti-rabbit IgG diluted 1:400 was added and incubated at RT for 45 min. After rinsing the sample twice with 1× PBS, 1:400 dilution of Rhodamine Phalloidin was added to stain the cell actin cytoskeleton for 30 min, then a 1:2000 dilution of DAPI was used to counter-stain the nucleus. After rinsing 3 times with 1 × PBS, the samples were imaged with CLSM. The CD31 staining positive area was averaged from 6 random fields of each cell scaffold sample (*n* = 4 samples per group) with the software ImageJ.

### 2.8. Co-Culture In Vitro

To demonstrate that endothelialization is necessary for the growth and structural remodeling of vascular SMCs, we successively inoculated mouse PAECs and SMCs into the inner and outer layers of HA/collagen nanofiber scaffolds. In short, HA/collagen nanofiber tube scaffold (2.5 cm length, 3 mm inner diameter) was inserted in a 3.5 cm cell culture plate, 70% ethanol solution was added to sterilize the scaffold at RT for 2 h and finally, pre-warmed 1× PBS was added to wash the scaffold 3 times. The sterilized tube scaffold was closed at both ends by sterile 5–0 sutures (VCP359H, Johnson and Johnson, New Brunswick, NJ, USA) to prevent cells from leaking out. PAECs were injected into the lumen at a density of 1 × 10^6^ cells/mL via a 25 G needle (SYRINGE, Jiangyin Fanmei Medical Device Co, Ltd., Jiangyin, China). The cell scaffold was transferred to a 15 mL test tube containing 10 mL of culture medium and rotated at a speed of 10 rpm in an incubator at 37 °C. After 24 h, the suture was removed; the cell scaffold was transferred to a 3.5 cm cell culture plate, and incubated at 37 °C and 5% CO_2_ for 3 days. Then, the mouse aortic SMCs were seeded into the outer layers of the scaffolds at a density of 1 × 10^7^ cells/cm^2^ at 37 °C for 24 h. The cell scaffold was rotated 45 degrees to seed the SMCs into the outer layers of the scaffolds at the above-mentioned density at 37 °C for 24 h, continuously until the outer layer of the tubular scaffold was inoculated with SMCs. After 5 days of co-cultivation, the cell scaffold samples were collected and fixed in 4% paraformaldehyde for 15 min. Then, the samples were embedded with optimum cutting temperature compound (OTC, SAKURA, Torrance, CA, USA) and frozen sections cut at 15 μm. Cell scaffolds were permeabilized with 01% Triton X-100 in 1× PBS for 10 min and blocked with 1% BSA in 1× PBS for 30 min at RT.

The following antibodies, mouse anti-CD31 monoclonal antibody and rabbit anti-α-SMA polyclonal antibody, were used at a dilution of 1:150. Sections were then incubated for 45 min at RT with species-matched Cy3 or Alexa Fluor-488-conjugated secondary antibodies. Following specimen washing with 1× PBS, nuclei were counterstained with DAPI. Then, samples were imaged with CLSM. Histomorphometric evaluations (*n* = 3 samples per group) were performed on three independent microscopic fields (20 × magnification) using ImageJ software (http://imagej.net/citing) (accessed on 22 April 2017) to quantify CD31 and α-SMA positivity.

The same cross-sections were stained with H&E, MTC and VVG using standard methods. Images of stained samples were performed with a Pannoramic DESK microscope. ImageJ software was employed to convert the blue colour intensity of the MTC stains, relative to collagen composition, in mean gray value with respect to tissue area. This protocol used hue (121–179), saturation (20–255) and brightness (10–255) to isolate collagen, also adaptable for elastin and muscle content. Both muscle content and elastin were identified using the YUV color space (muscle: Y = 0–145, elastin: Y = 0–120, both: U and V = 0–255).

### 2.9. Statistical Analysis

Where appropriate, a one-way or two-way analysis of variance (ANOVA) was performed using Graphpad Prism 7.0 software to determine the significant difference of the Tukey post hoc test (*p* < 0.05). Unless otherwise stated, data and error bars are reported as mean × standard deviation.

## 3. Results and Discussion

### 3.1. Preparation and Characterization of HA/Collagen Nanofibers

**Figure 1:**
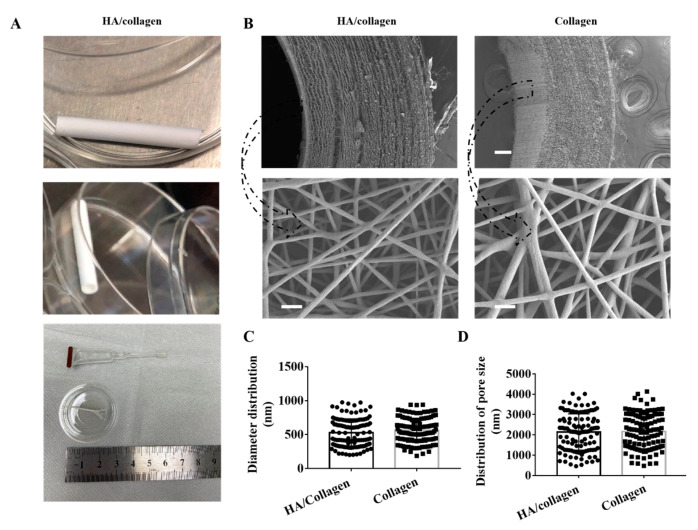
Physical characteristics of electrospun nanofibers. (**A**) The lateral (upper panel), cross-section (middle panel) and strip nanofiber concentric axis film (after cutting along the axis) in the image of an HA/collagen nanofiber tube. (**B**) SEM micrographs of the cross-section (upper panel) views and the inner wall surface (lower panel) of HA/collagen and collagen nanofibers before cross-linking. Scale bars: 100 µm (upper panel), 2 µm (lower panel). Statistical data of the diameter (**C**), pore size, and (**D**) distribution of various nanofibers.

The tubular HA/collagen nanofibrous vessel scaffolds with hierarchical architecture were fabricated with HA and collagen by sequential electrospinning. The tube has an inner diameter of 3.0 mm and a wall thickness of ~0.62 mm (Figure 1A). The inner wall of the tube is a layer of pure HA nanofibers with a thickness of ~0.22 mm, and the outer wall is a layered pure collagen nanofibrous structure with a thickness of ~0.4 mm (Figure 1B). SEM micrographs showed that the inner wall surface of electrospun HA/collagen and collagen nanofiber tubes possesses a porous nanofiber network-like nano-topography (Figure 1B). The single nanofibers are in a continuous cylindrical shape. Quantitatively, the average nanofiber diameter of an HA/collagen nanofiber tube is (541.3 ± 19.3) nm, which is slightly smaller than that (580 ± 16) nm of pure collagen nanofiber, but not statistically significant (Figure 1C). The average pore diameter of the inner wall of the HA/collagen and pure collagen nanofiber is (2139 ± 81) and (2159 ± 77) nm, respectively (Figure 1D). HA was physically immobilized on the inner surface of the hollow tubular collagen scaffold, rather than covalently linked, which may change the activity of HA. Different sizes of vascular scaffolds were obtained by adjusting the mold (receiving device). As a proof of concept, this procedure can be applied for other hollow organs. The inner diameter of the scaffold is 3 mm to simulate the small diameter artery. By adjusting the mold of the receiving device, it can easily adapt to the size and diameter for specific patients.

**Figure 2:**
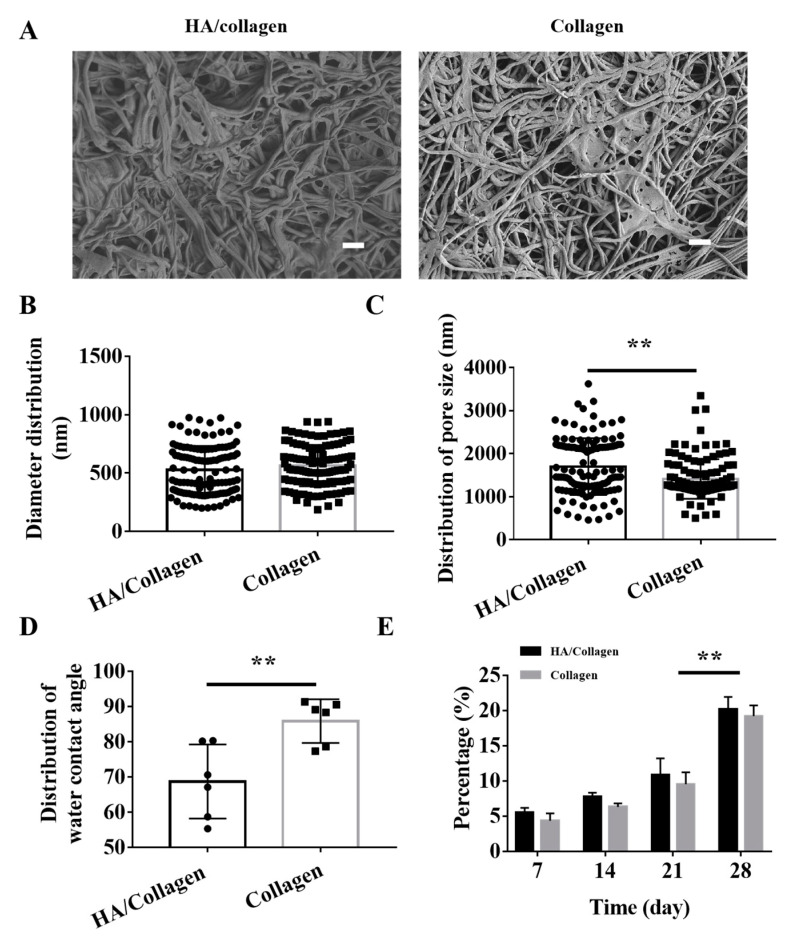
Morphology, hydrophilicity and degradability of cross-linked electrospun nanofibers in vitro. (**A**) SEM micrographs of the surface of cross-linked electrospun HA/collagen and collagen nanofibers. Scale bars: 4 μm. Statistical data of the diameter (**B**) and pore size (**C**) distribution of cross-linked nanofibers (*n* = 120). Average water contact angle (**D**) and degradability (**E**) of different nanofibers (*n* = 6). ** *p* < 0.01.

HA is a hydrophilic bioactive macromolecule [19,34]. To prevent the HA nanofibers on the inner wall of the tube from swelling when the HA/collagen nanofiber tube was used under physiological conditions, we used glutaraldehyde/acetone treatment to induce cross-linking between molecules of HA and collagen. SEM morphology observation showed that electrospun HA/collagen and collagen nanofibers formed a tight interconnected nanofiber porous structure under the effect of glutaraldehyde/acetone crosslinking (Figure 2A). After cross-linking, the average nanofiber diameters of HA/collagen and collagen nanofibers were (527.3 ± 18) and (564 ± 16) nm, respectively (Figure 2B). The average pore size on the inner wall was (1699 ± 60) and (1411 ± 42) nm (Figure 2C). This indicates that crosslinking treatment has little effect on the average diameter of electrospun nanofibers, but has a significant effect on the average pore diameter of the electrospun nanofiber network structure.

The averaged static water contact angle of the inner surface of the crosslinked HA/collagen nanofibers is (68.7 ± 4.3)°, which is significantly smaller than the (85.9 ± 2.5)° of the pure collagen nanofiber tube (Figure 2D, data collected in Table S1). Due to the similar topography of electrospun HA/collagen and collagen nanofibers, the improved wettability of HA/collagen nanofibers can be attributed to the hydrophilic effect of HA.

The chemical properties of crosslinked collagen and HA/collagen nanofibers were detected by ATR-FTIR (Figure S1). The characteristic peaks of collagen nanofibers can be detected at 1656, 1556 and 1242 cm^−1^, corresponding to amide I, amide II and amide III, respectively, which are consistent with a previous report [19]. The peaks show that crosslinking is not causing changes in the secondary structure of peptides and proteins. Compared with collagen nanofibers, the peaks of HA/collagen nanofibers in amide I, amide II and amide III absorption were 1658, 1552 and 1239 cm^−1^, respectively. These changes may be due to the interaction between the aldehyde group of HA and the amine group of collagen. Figure S2 shows the XPS analysis of collagen and HA/collagen nanofibers detecting C, O, and N elements on their surface layers (Figure S2A), as expected from collagen-based on amino acids and HA, higher polysaccharide, composed of D-glucuronic acid and N-acetylglucosamine. In addition, a small amount of N1s (399.75 eV) signal appeared on the surface of collagen nanofibers, which was attributed to the amino group in collagen molecules (Figure S2B). After HA nanofibers covered the surface of collagen nanofibers, the content of N on the surface of HA/collagen nanofibers further increased (Figure S2B). The results show that HA nanofibers are successfully introduced into the surface of collagen nanofibers. The XRD patterns of HA/collagen and collagen nanofiber films are shown in Figure S3. The two nanofiber films show the characteristics of X-ray diffraction patterns of some crystalline materials. Collagen nanofiber film has a defined diffraction peak, and a wide peak at 2θ of 25.6°. This corresponds to the diameter of the triple helix collagen molecule. Compared with collagen nanofiber, the diffraction spectrum profile of the HA/collagen nanofiber film is similar, and the maximum peak intensity changes slightly, indicating that addition of HA has no effect on the crystallinity of collagen nanofiber film.

To determine the stability of the HA/collagen nanofiber tube structure, we tested the resistance of these electrospun fibers to plasminogen (an important enzyme in the blood that keep blood vessels and glandular ducts unobstructed). As shown in Figure 2E, by the seventh day, the weight loss rates of HA/collagen and collagen nanofiber tubes were (5.5 ± 0.7)% and (4.3 ± 1.1)%, respectively. By the 14th and 21st days, the weight loss rate of each sample increased slightly, but it was not statistically significant. As the experiment time was extended to the 28th day, the weight loss rates of HA/collagen and collagen nanofiber tubes were significantly greater than that on the 21st day. The weight loss rate of HA/collagen nanofiber tubes was 20.8%, slightly higher than 19.2% of collagen. These data indicate that HA/collagen nanofiber tubes have certain resistance to degradation in human plasma plasmin aqueous solution.

### 3.2. Mechanical Properties

**Figure 3:**
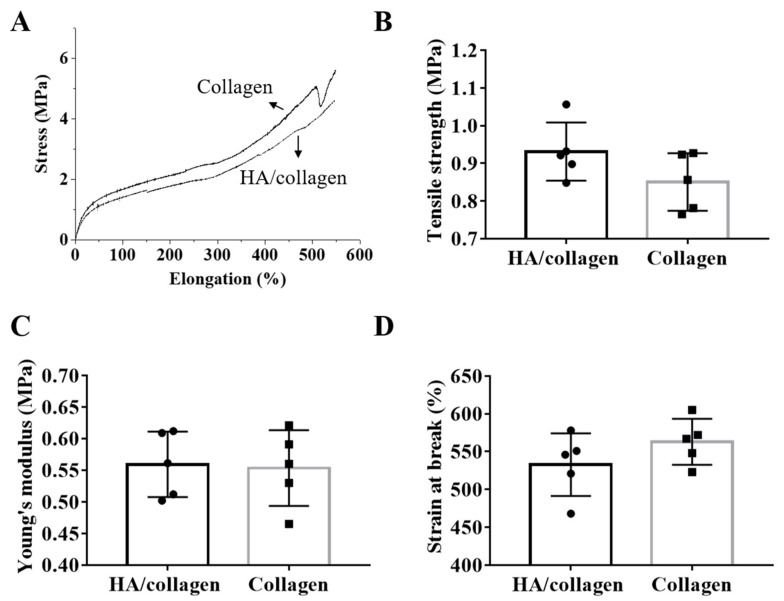
Mechanical properties. Relative to cross-linked electrospun HA/collagen and collagen nanofibers: (**A**) typical stress–strain curves, (**B**) ultimate tensile strength, (**C**) Young’s modulus and (**D**) strain at break.

To analyze the mechanical properties of the scaffold, each electrospun nanofiber film was tested in uniaxial tension mode to generate a stress–strain curve and derived tensile properties (Figure 3A). HA/collagen nanofiber films showed an ultimate tensile strength of (0.97 ± 0.1) MPa (Figure 3B), Young’s modulus of (0.56 ± 0.02) MPa (Figure 3C) and elongation at break of (532.8 ± 18.62)% (Figure 3D), respectively collected in Table S1. Although the Young’s modulus is equivalent to collagen, the ultimate stretch of collagen nanofibers is slightly lower than that of HA/collagen nanofibers, but not statistically significant. Both HA/collagen and collagen nanofibers have relatively high elongation at break (Figure 3A,D), indicating that both specimens are very soft and tough materials, exhibiting good ductility after stretching. These data suggest that the introduction of HA does not affect the mechanical properties of collagen. The mechanical properties of HA/collagen nanofiber tubes are derived from collagen nanofibers, while HA fixed on the inner wall surface of HA/collagen scaffold is helping to optimize the surface biochemistry. In previous studies we showed that mimicking porosity and mechanical properties of natural ECM promotes tissue regeneration [35].

### 3.3. Hemocompatibility of the HA/Collagen Nanofibers In Vitro

**Figure 4:**
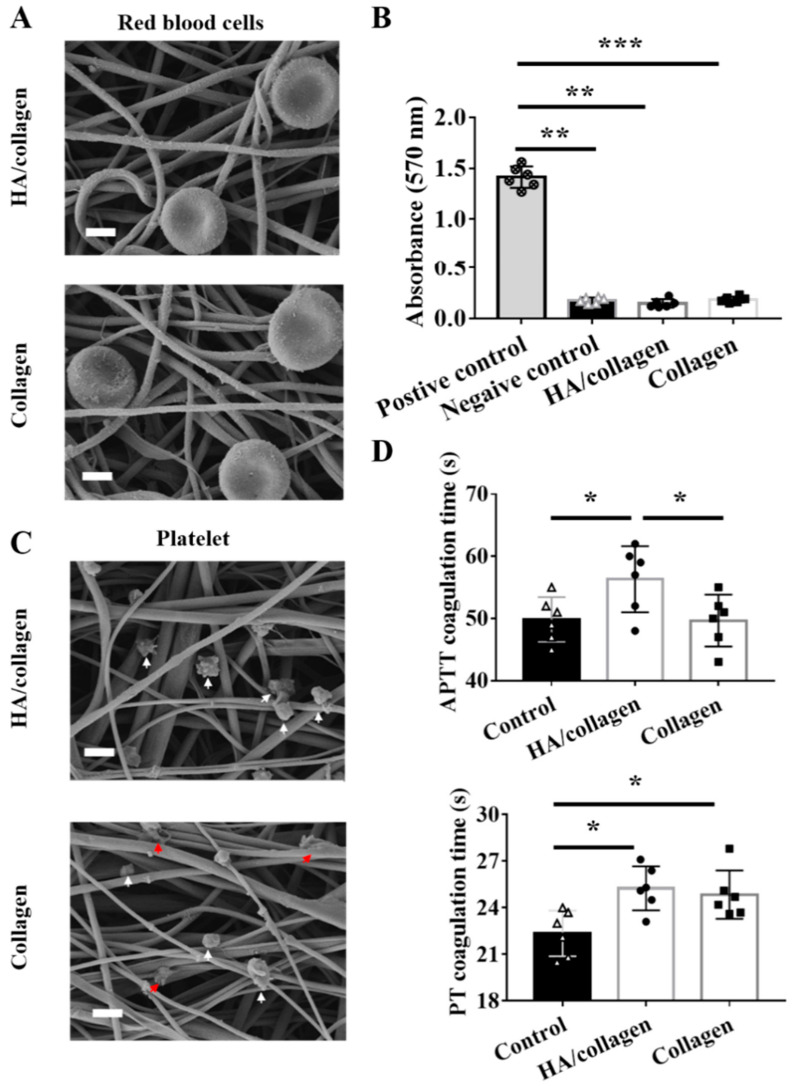
Hemolysis and anticoagulation tests. Representative SEM micrographs of red blood cells (**A**) and platelets (**C**) on HA/collagen and collagen nanofibers after 2 h seeding. White arrows indicate non-adherent platelets, red arrows indicate adherent platelets. Scale bars: 1 µm. Hemolysis rate (**B**) of different nanofibers. Statistical data of APTT and PT (**D**) of control native plasma and plasma incubated with different nanofibers. *n* = 3, * *p* < 0.5; ** *p* < 0.01; *** *p* < 0.001.

The morphology and lysis of RBCs after 1 h incubation on HA/collagen and collagen nanofibers were observed by SEM (Figure 4A). On different substrates, the shape of the RBC is a biconcave disk with a diameter of about 7–8 μm, with a concave central part (about 1 μm thick) and thick edges (about 1.9 μm thick). No morphological changes or cracks were observed. Furthermore, the hemoglobin released from the red blood cells was measured using a colorimetric method to determine the hemolysis rate. As shown in Figure 4B, according to the ISO 10993-4:2002 standard and ASTM F756-00 (2000), all nanofibers are non-hemolytic and will not cause any detectable hemolysis.

The antithrombotic properties of electrospun nanofiber scaffolds were evaluated by in vitro platelet adhesion measurement. The platelet adhesion of platelet-rich plasma (PRP) after 1 h incubation on different nanofiber substrates was observed by SEM (Figure 4C). A small amount of platelets was observed on the network structure of HA/collagen nanofibers, the shape was round, oval or irregular, and the diameter was about 1.5 μm. In contrast, on collagen nanofibers, some of the adherent platelets are activated, and the shape of the prosthetic foot is flat and extended.

Moreover, we evaluated the clotting time by calculating APTT to observe the influence of the material on the delay of clotting through the internal pathway [36]. The results showed that, compared with the control, the APTT value of HA/collagen nanofibers was (56.3 ± 2.2) s (*p* < 0.05), while the APTT value of collagen was (49.7 ± 1.7) s (Figure 4D, *p* < 0.05). In addition, we also tested the PT of each electrospun fiber to evaluate the material’s effect on coagulation in the external pathway of the coagulation cascade [19]. Compared with the control group, the PT values of HA/collagen and collagen nanofibers increased significantly (Figure 4D, *p* < 0.05). These results indicate that the introduction of HA can improve the anticoagulant activity of the substrate.

### 3.4. HA/Collagen Nanofibers Can Promote Complete Endothelialization of Vascular EC

**Figure 5:**
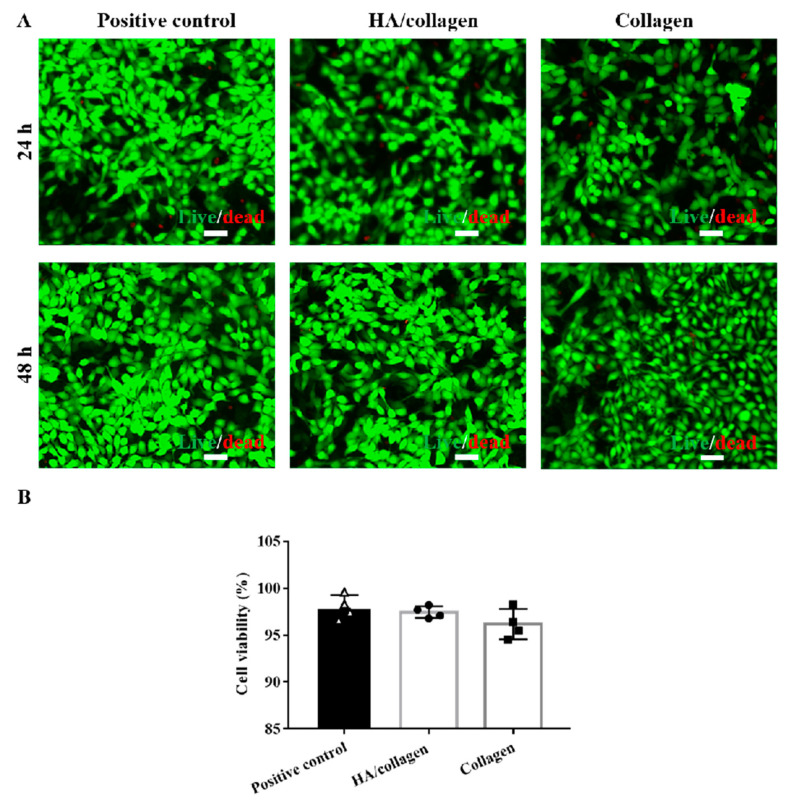
In vitro cytocompatibility analysis. (**A**) Fluorescence microscopy graphs of stained mouse PAECs with and without crosslinked nanofiber films after 24 and 48 h of culture. Scale bars, 50 µm. (**B**) The cell viability of mouse PAECs with and without crosslinked nanofiber films after 48 h of culture.

Before measuring cell growth behavior, we evaluated the cytotoxicity of collagen and HA/collagen nanofibers by cell Live/dead staining. As shown in Figure 5, PAECs were co-cultured with collagen and HA/collagen nanofiber films for 48 h. The results showed that collagen and HA/collagen nanofiber films did not demonstrate obvious cytotoxicity to PAECs after 48 h of co-culture, and the cell viability was (96.4 ± 1.7)% and (98.2 ± 0.5)%, respectively. Since the measured cell viability is greater than 95%, it can be considered that cross-linked collagen and HA/collagen nanofibers can be used as vascular scaffolds [19,20].

**Figure 6:**
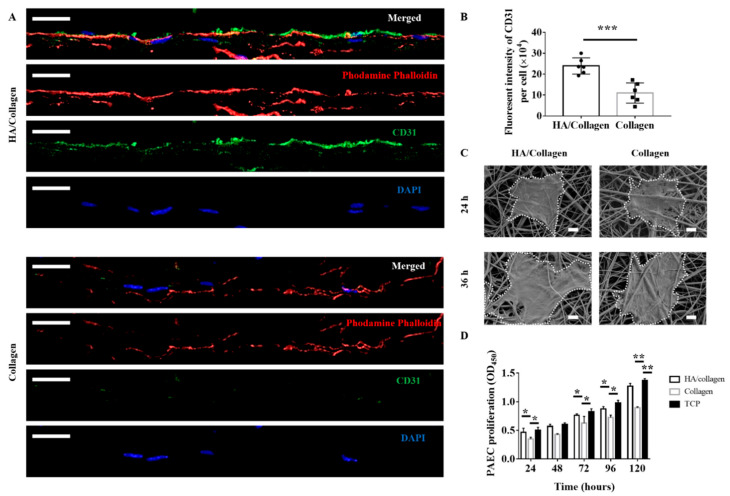
In vitro phenotypic shape and proliferation analysis of mouse PAECs on different substrates. Representative CLSM micrograph cross-section (**A**) of PAECs on different nanofibers after 72 h seeding. Nucleus (blue), cytoskeleton (red), CD31 (green). Scale bars: 20 μm. Statistical data of CD31 fluorescence (**B**) in ECs after 72 h seeding (*n* = 5). *** *p* < 0.001. Representative SEM micrographs (**C**) of PAECs on different nanofibers after 24 and 36 h seeding. Scale bars: 5 µm. The proliferation (**D**) of PAECs on different nanofibers and TCP (positive control) during a period of 5 days (*n* = 3). * *p* < 0.5; ** *p* < 0.01.

Next, we studied the growth behaviour of PAECs grown on HA/collagen nanofibers. As shown in Figure 6A, 72 h after inoculation, the cytoskeleton (red fluorescence) of mouse PAECs on the surface of HA/collagen and collagen nanofibers was completely extended, indicating that intercellular junctions were basically established. The staining of platelet EC adhesion molecule-1 (CD31), a specific marker for vascular ECs [37], showed that the signals of CD31-positive cells (green fluorescence) exist in vascular ECs growing on the surface of HA/collagen nanofibers, while the CD31-positive signal was weak in vascular EC growing on the surface of collagen nanofibers. Quantitatively, the density of CD31+ signals on HA/collagen nanofibers was higher than that of collagen nanofibers (Figure 6B, *p* < 0.01). It is reported that increased expression of CD31 contributes to endothelialization, while decreased expression of CD31 indicates cell dysfunction [19]. The surface biochemistry and biophysical characteristics of biomaterial scaffold are the key factors of cell adhesion, growth, proliferation and differentiation [3,17,23]. For example, ECM stiffness can regulate the integrity, permeability and leukocyte migration of vascular EC monolayers [12,23,25,38,39]. Moreover, controlling nanotopography and mechanical properties can be used to stimulate cells by mimicking their natural environment, triggering the correct functionalities [40,41]. The vascular EC growth on exogenous HA or its oligomer functionalized polymer scaffolds showed improved phenotypic shape and proliferation behaviour [30]. Scaffolds loaded with soluble biochemical factors (e.g., vascular endothelial growth factor, VEGF) can promote the adhesion, migration, proliferation and differentiation of vascular EC [42,43]. In this study, the surface of HA/collagen nanofibers may be an ideal matrix for the rapid and complete endothelialization of PAECs.

We further used SEM to observe the morphology of PAECs at the early stage (24–36 h after seeding) on these two electrospun nanofiber substrates (Figure 6C). After 24 h, PAECs adhered to the surface of all nanofibers with varying degrees of elongation. By 36 h, the PAECs on the surface of HA/collagen nanofibers showed a more significant elongation, suggesting that the HA/collagen substrate is more beneficial to mouse PAEC elongation.

To determine whether electrospun HA/collagen nanofibers affect the proliferation of vascular ECs, we used CCK-8 to analyse the growth of mouse PAECs on different nanofiber substrates. As shown in Figure 6D, mouse PAECs can proliferate on HA/collagen nanofibers for more than 5 days. It should be noted that on day 5, the optical density (OD) value of mouse PAECs cultured on HA/collagen nanofibers was significantly higher than that of collagen nanofibers, and was close to those of mouse PAECs cultured on TCP. As pure collagen nanofibers could not promote the proliferation and phenotypic expression of PAECs, they were not used in subsequent studies. These results are consistent with previous reports showing that scaffold surface biophysical and biochemical properties are crucial for vascular EC functions [19,20,21,22,23].

### 3.5. Complete Endothelialization of Tubular HA/Collagen Nanofibers Dictating Vascular SMCs Infiltration and Alignment

**Figure 7:**
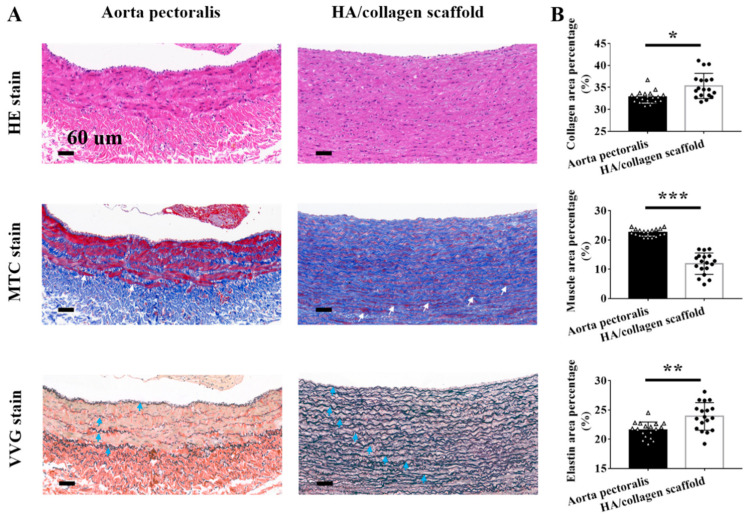
Histological evaluation. (**A**) Representative H&E, MTS, and VVG staining of rabbit native aorta pectoralis and cellularized tubular HA/collagen scaffold. Scale bars: 60 µm. (**B**) Statistical data of collagen, muscle bundles and elastin of rabbit native aorta pectoralis and cellularized tubular HA/collagen scaffolds (*n* = 3). * *p* < 0.5; ** *p* < 0.01; *** *p* < 0.001.

A healthy and confluent EC lining is essential for the structural reconstruction of vascular wall tissue damaged by vascular injury or disease [3,39,44,45,46,47]. To verify whether the endothelialization of the inner wall surface of the tubular scaffold plays a beneficial role in the subsequent spatial distribution of vascular SMCs, we first seeded PAECs on the inner surface of HA/collagen nanofibers to form an endothelialized ECs monolayer, and then SMCs were seeded on the outer surface of HA/collagen nanofibers. After 5 days of coculturing in vitro, the distribution of ECs and SMCs in the cellularized scaffolds was observed. As shown in Figure 7A, H&E staining showed that PAECs lined the inner wall luminal surface of the tube, forming a thin layer of flat epithelium, arranged closely. The nucleus is in the middle and slightly protruding. The basal surface of the cell adheres to the inner basal plate. The morphology of PAECs was similar to that of ECs in mouse aortic endothelium (Figure 7A). SMCs adhered to and grew into the wall under the PAEC layer. They were evenly distributed in layers and showed a wavy shape, similar to the shape of natural arteries. MTS staining on the same cross-section showed that PAECs and SMCs on the cross-section of HA/collagen scaffold could synthesize collagen (blue), and the collagen nanofibers were curly and wavy, and evenly distributed around PAECs and SMCs. At this time, a small number of smooth muscle bundles (red) were formed under the EC layer. VVG staining of the same cross-section showed that both PAECs and SMCs on the cross-section of HA/collagen scaffold could synthesize elastin (black) in a wavy shape. Quantitatively, the contents of collagen nanofibers, smooth muscle bundles and elastin in the tissue-engineered vascular grafts constructed by 5 days of culture in vitro were significantly lower than those in the native arteries (Figure 7B). These findings suggest that the bulk hierarchical architecture of HA/collagen scaffolds directs the spatial distribution of PAECs and SMCs as well as the synthesis of ECM.

**Figure 8:**
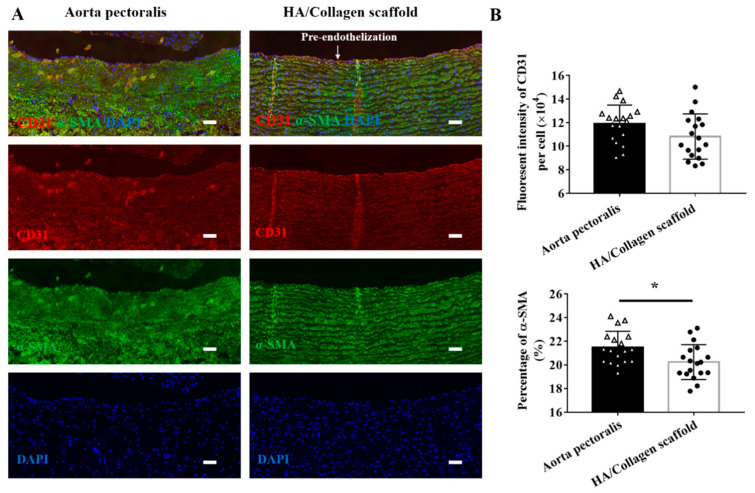
Fluorescence immunostaining. (**A**) Representative CLSM micrographs of rabbit native aorta and cellularized tubular HA/collagen scaffold. Nucleus (blue), CD31 in vascular EC (red), α-SMA in vascular SMC (green). Scale bars: 60 μm. (**B**) Statistical data of CD31 and ɑ-SMA fluorescence of rabbit native aorta and cellularized tubular HA/collagen scaffolds. * *p* < 0.5.

In addition, we also studied the phenotypic shape of PAECs and SMCs on tubular HA/collagen nanofiber scaffolds. The PAECs and SMCs on the inner wall and outer wall surfaces were labelled with anti-mouse CD31-Cy3 and anti-rabbit α-SMA (a SMC specific marker [23])-Alexa staining, respectively (Figure 8A). The CLSM images show that a thin layer of CD31 positivity (red fluorescence) is evenly distributed on the inner wall of HA/collagen nanofiber tubes, and the contractile protein α-SMA positive (green fluorescence) of SMCs is restricted under the EC layer and evenly distributed. The characteristics of endothelium and smooth muscle are close to those of a native artery. Quantitatively, the content of CD31 positivity and α-SMA positivity was slightly lower than that of a native artery (Figure 8B). On the contrary, the α-SMA-positive (green fluorescence) signal in vascular SMCs grown on non endothelialized tubular HA/collagen nanofiber scaffolds was very weak, and vascular SMCs preferentially gathered on the inner surface of tubular HA/collagen scaffolds (Figure S4). Therefore, complete endothelialization may determine the phenotypic shape and growth pattern of vascular SMCs on tubular HA/collagen scaffolds.

## 4. Conclusions

Electrospinning technology was used to prepare a hierarchical architecture nanofiber scaffold based on HA and collagen, which can temporarily create an environment similar to native artery tissue. Thin HA nanofibers were fixed on the inner surface of the tubular nanofibers, so that the resultant HA/collagen nanofibers showed anisotropic wetting behaviour and mechanical compliance, as required for vascular scaffolds. Primary vascular ECs cultured on HA/collagen nanofibers showed the phenotypic shape and proliferation ability ideal for complete endothelialization, which may be directed by the synergistic effect of biochemical and biophysical cues of scaffolds. Most importantly, the complete endothelialization of vascular ECs along the inner wall of HA/collagen nanofibers also facilitate vascular SMC infiltration and alignment along their hierarchical architecture. In turn, SMCs facilitate the maintenance of their contractile phenotypic shape as well as establish natural growth behaviour patterns. In addition, HA/collagen nanofibers have good stability and blood compatibility, highlighting their suitability for tissue engineering vascular implants. Future perspectives of this work may involve in vivo studies to evaluate the performance of cellularized vascular stents. If this scaffold is durable enough after implantation in animals, cell scaffolds constructed from human cells may be used as living vascular prostheses for small caliber arteries. Our vascular stent is attractive for this application because it is lined with functional endothelium and can heal at the anastomosis, truly integrating with the host vascular system.

## Data Availability

The data presented in this study are available on request from the corresponding authors.

## Supplementary Materials

The following are available online at https://www.mdpi.com/2079-4991/11/9/2334/s1, Figure S1: ATR-FTIR spectra collected for crosslinked collagen and HA/collagen nanofiber films, Figure S2: Surface chemical element distribution of collagen and HA/collagen nanofiber films after crosslinking treatment., Figure S3: XRD pattern of collagen and HA/collagen nanofiber film surfaces after crosslinking treatment. Figure S4: Fluorescence immunostaining of cross-section of cellularized scaffold, Table S1: Contact water angles and mechanical properties of HA/collagen and Collagen nanofibers.
