# Correction: Niu, Y.; Galluzzi, M. Hyaluronic Acid/Collagen Nanofiber Tubular Scaffolds Support Endothelial Cell Proliferation, Phenotypic Shape and Endothelialization. *Nanomaterials* 2021, *11*, 2334

**DOI:** 10.3390/nano14141203

**Published:** 2024-07-16

**Authors:** Yuqing Niu, Massimiliano Galluzzi

**Affiliations:** 1 Department of Pediatric Surgery, Guangdong Provincial Key Laboratory of Research in Structural Birth Defect Disease, Guangzhou Women and Children’s Medical Center, Guangzhou Medical University, Guangzhou 510623, China; 2 Materials Interfaces Center, Shenzhen Institutes of Advanced Technology, Chinese Academy of Sciences, Shenzhen 518055, China; galluzzi@siat.ac.cn

## Error in Figures

In the original publication [1], there were mistakes in Figure 1B and Figure 2A. The SEM micrograph of the cross-section view of the HA/collagen sample before cross-linking in the upper panel of Figure 1B was erroneously displayed for a wall thickness of ~0.35 mm, and the correct image was originally omitted. In Figure 2A, the SEM micrographs of the surface of cross-linked, electrospun HA/collagen nanofibers and collagen nanofibers samples were inadvertently displayed before cross-linking images during the figure arrangement. The corrected upper panel for Figure 1B and the corrections for Figure 2A are shown below.

In the published version of Figure 5A, the fluorescence micrograph of the collagen sample in the right lower panel in Figure 5A is incorrectly represented. The corrected figure is displayed below.

The authors apologize for any inconvenience caused and state that the scientific conclusions are unaffected. This correction was approved by the Academic Editor. The original publication has also been updated

## Figures and Tables

**Figure 1:**
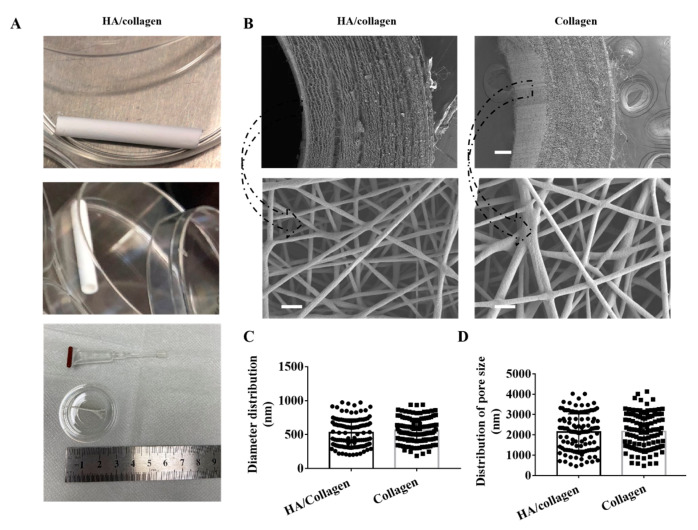
Physical characteristics of electrospun nanofibers. (**A**) The lateral (upper panel), cross-section (middle panel) and strip nanofiber concentric axis film (after cutting along the axis) in the image of an HA/collagen nanofiber tube. (**B**) SEM micrographs of the cross-section (upper panel) views and the inner wall surface (lower panel) of HA/collagen and collagen nanofibers before cross-linking. Scale bars: 100 µm (upper panel), 2 µm (lower panel). Statistical data of the diameter (**C**), pore size, and (**D**) distribution of various nanofibers.

**Figure 2:**
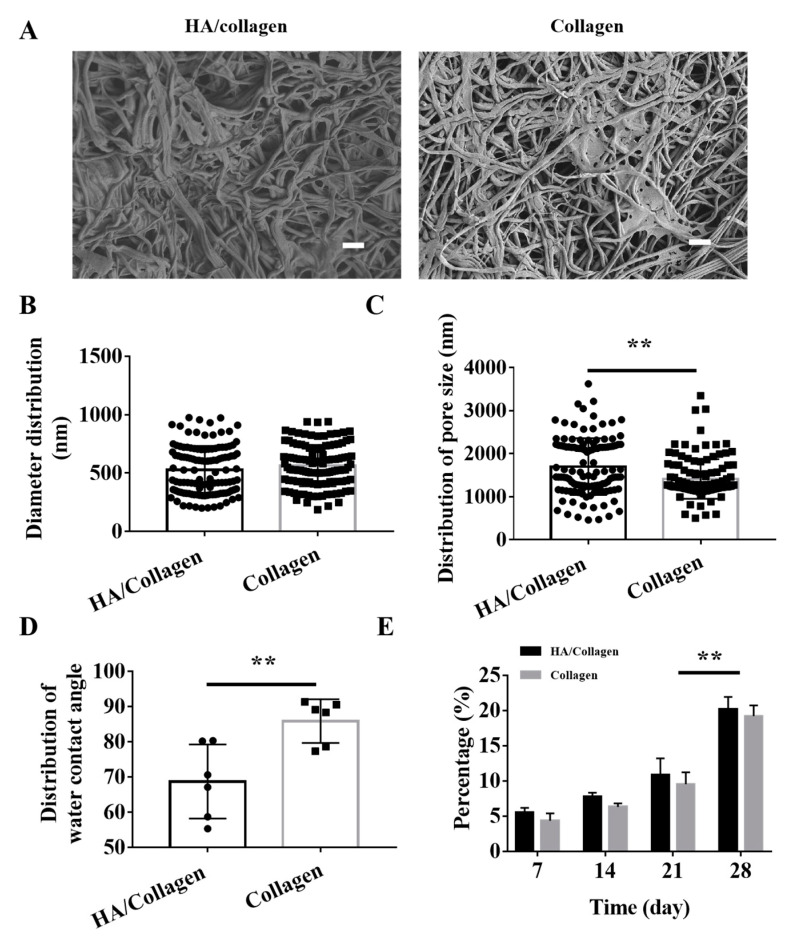
Morphology, hydrophilicity and degradability of cross-linked electrospun nanofibers in vitro. (**A**) SEM micrographs of the surface of cross-linked electrospun HA/collagen and collagen nanofibers. Scale bars: 4 µm. Statistical data of the diameter (**B**) and pore size (**C**) distribution of cross-linked nanofibers (*n* = 120). Average water contact angle (**D**) and degradability (**E**) of different nanofibers (*n* = 6). ** *p* < 0.01.

**Figure 5:**
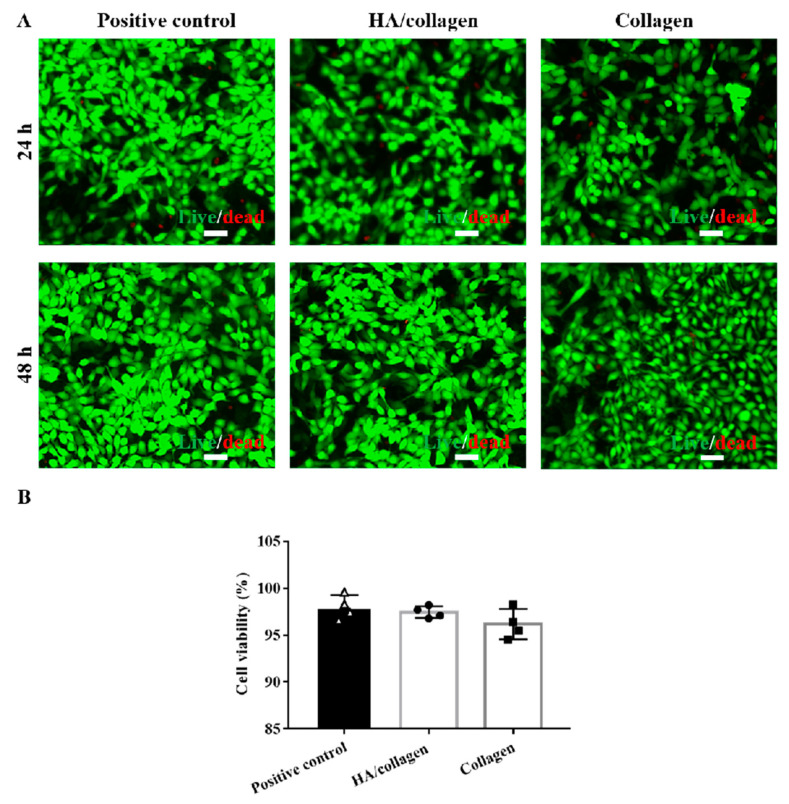
In vitro cytocompatibility analysis. (**A**) Fluorescence microscopy graphs of stained mouse PAECs with and without crosslinked nanofiber films after 24 and 48 h of culture. Scale bars, 50 µm. (**B**) The cell viability of mouse PAECs with and without crosslinked nanofiber films after 48 h of culture.
