# Supplementary material for: Hyaluronic Acid/Collagen Nanofiber Tubular Scaffolds Support Endothelial Cell Proliferation, Phenotypic Shape and Endothelialization
Source: Nanomaterials (Basel). 2021 Sep 8;11(9):2334. doi: 10.3390/nano11092334 (PMC8471775; doi:10.3390/nano11092334)
Supplement: Supplementary file 1 [file nanomaterials-11-02334-s001.zip › nanomaterials-1296318-supplementary-proof-latest.pdf]

nanomaterials

MDPI

Supplementary Materials

Hyaluronic Acid/Collagen Nanofiber Tubular Scaffolds Support Endothelial Cell Proliferation, Phenotypic Shape and Endothelialization

Yuqing Niu [1,*] and Massimiliano Galluzzi [2]

[1] Department of Pediatric Surgery, Guangdong Provincial Key Laboratory of Research in Structural Birth Defect Disease, Guangzhou Women and Children's Medical Center, Guangzhou Medical University, Guangzhou 510623, Guangdong, China
[2] Materials Interfaces Center, Shenzhen Institutes of Advanced Technology, Chinese Academy of Sciences, Shenzhen 518055, China; galluzzi@siat.ac.cn
* Correspondence: niuyuqing@gwcmc.org

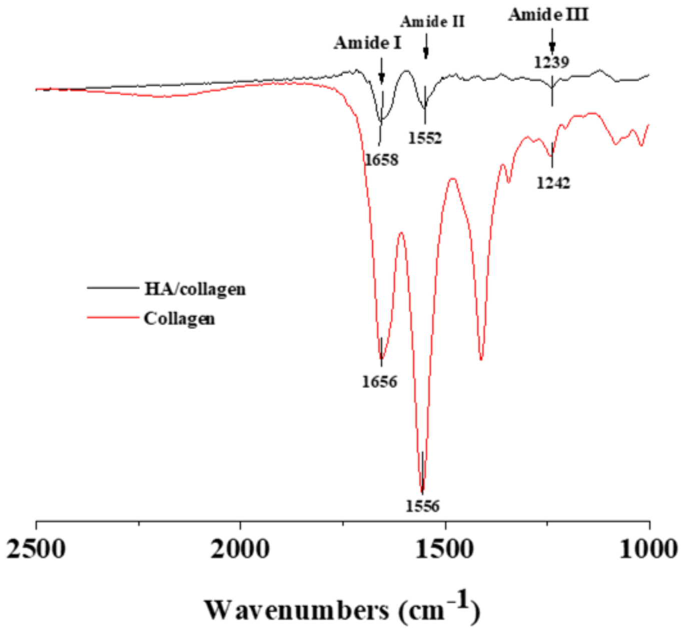

Figure S1. ATR-FTIR spectra collected for crosslinked collagen and HA/collagen nanofiber films.

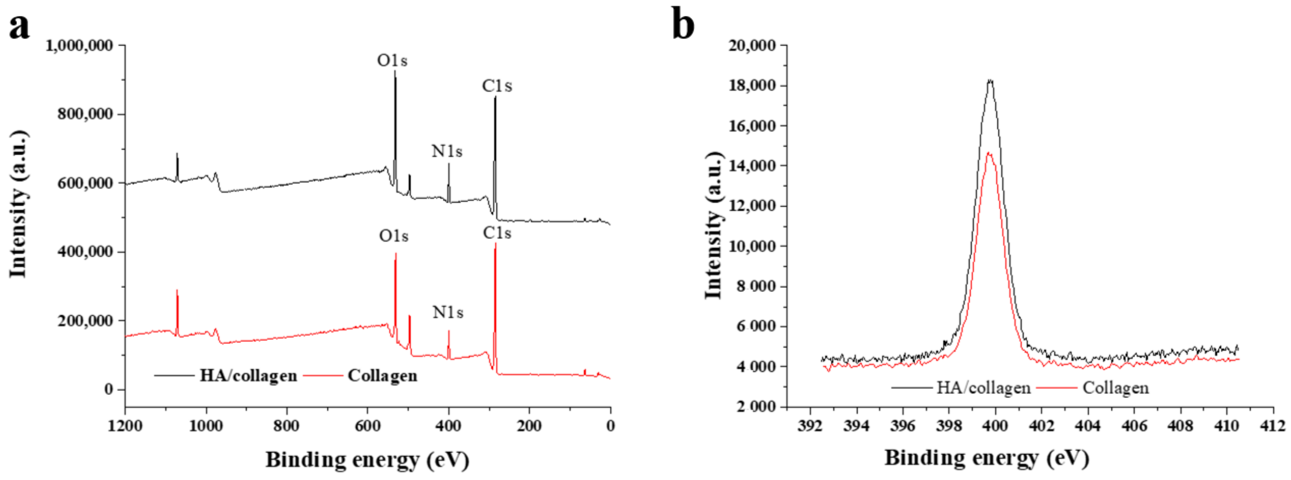

Figure S2. Surface chemical element distribution of collagen and HA/collagen nanofiber films after crosslinking treatment. (**A**) XPS spectrum of O, N, and C peaks, (**B**) N1s curve fitting at high-resolution.

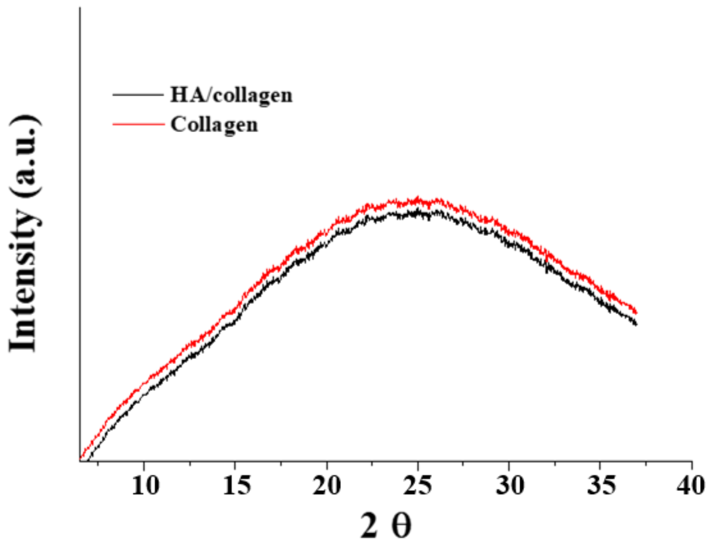

Figure S3. XRD pattern of collagen and HA/collagen nanofiber film surfaces after crosslinking treatment.

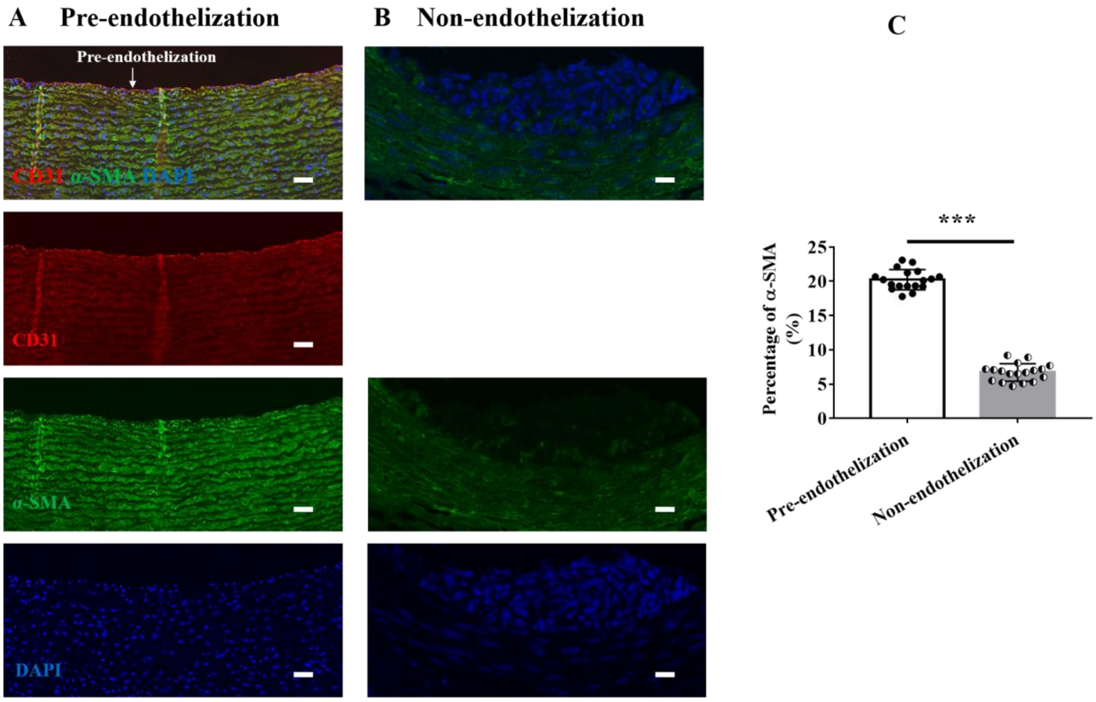

Figure S4. Fluorescence immunostaining of cross-section of cellularized scaffold. Representative CLSM micrographs of tubular HA/collagen scaffolds of (**A**) pre-endothelization and (**B**) non-endothelization. Scale bars: 60 μm. (**C**) Statistical data of α-SMA fluorescence of cellularized tubular HA/collagen scaffolds. ***$p < 0.001$.

Table S1. Contact water angles and mechanical properties of HA/collagen and Collagen nanofibers.

Sample	θ H2O (°)[a]	E (MPa)[b]	δ (MPa)[c]	ε (%)[d]
**HA/collagen**	68.7±4.3	0.56±0.02	0.93±0.03	532.8±18.6
**Collagen**	85.9±2.5	0.55±0.03	0.85±0.04	563±13.6

[a]: Contact water angle.
[b]: Young's modulus.
[c]: Stress at yield.
[d]: Strain at break.
